# Serum Spot 14 concentration is negatively associated with thyroid-stimulating hormone level: Erratum

**DOI:** 10.1097/MD.0000000000005659

**Published:** 2016-12-09

In section “2.5 Statistical Analysis” of the article “Serum Spot 14 concentration is negatively associated with thyroid-stimulating hormone level”,^[1]^ which appeared in Volume 95, Issue 40 of *Medicine*, data for Group 2 was originally presented incorrectly. The data for Group 2 should have appeared as follows: Group 2: 0.004 < TSH ≦ 0.422.

## References

[1] Chen Y-T Tseng F-Y Chen P-L Serum Spot 14 concentration is negatively associated with thyroid-stimulating hormone level. *Medicine*. 2016 95:e5036. 27749565 10.1097/MD.0000000000005036 PMC5059067

